# Awareness and Knowledge of Physical Activity Recommendations in Young Adult Men

**DOI:** 10.3389/fpubh.2019.00310

**Published:** 2019-10-30

**Authors:** Jani P. Vaara, Tommi Vasankari, Harri J. Koski, Heikki Kyröläinen

**Affiliations:** ^1^Department of Leadership and Military Pedagogy, National Defense University, Helsinki, Finland; ^2^UKK Institute of Health Promotion Research, Tampere, Finland; ^3^Finnish Defence Forces, Helsinki, Finland; ^4^Faculty of Sport and Health Sciences, University of Jyvaskyla, Jyväskylä, Finland

**Keywords:** knowledge, awareness, aerobic physical activity, muscular type of physical activity, physical activity guidelines

## Abstract

**Background:** There are only a few studies that have assessed awareness and knowledge regarding aerobic type of physical activity (PA) guidelines recommendations, while no previous studies have examined muscular type of activity recommendation guidelines. The aim was to assess knowledge and awareness and study the associations with demographic variables and physical activity and fitness.

**Methods:** Participants were 776 young (age 26 ± 7 years) men. Awareness and knowledge of PA recommendation guidelines were assessed by prompted questionnaires together with demographic variables and physical activity. In addition, physical fitness was measured.

**Results:** Forty percent of the participants reported being aware of the physical activity recommendation. Moreover, 7% correctly identified the recommendations for moderate aerobic physical activity and 25% for muscular type of activity. In addition, 4% correctly identified both aerobic and muscular activity recommendations. Being aware of the PA recommendations was associated with being married or partnered, having higher education level and being more physically active during leisure-time (*p* < 0.05). Individuals with no awareness of the recommendations had lower results in cardiorespiratory and muscular fitness compared to those being aware (*p* < 0.05). Being married or partnered was positively associated with the knowledge of the muscular activity recommendations (*p* < 0.05). Moreover, the individuals with correct knowledge of the PA recommendations had higher levels of muscular fitness (*p* < 0.05).

**Conclusion:** A low number of individuals are aware or know the physical activity recommendations among young adult men. Therefore, more vigorous attempts to promote physical activity recommendations are needed.

## Introduction

Physical activity (PA) is an important factor in health promotion and disease prevention, and physical inactivity is linked with many chronic diseases and their risk factors (1). Therefore, PA recommendations have been established in order to give information to people of what is, in general, a minimum amount of PA to improve health. The aim is to increase knowledge, which would optimally to be reflected in PA behavior. The first PA recommendations were established in the publication of “*Guidelines for Graded Exercise Testing and Exercise Prescription”* in 1975 (2) and the recommendations were mostly aim to develop cardiorespiratory fitness. In 1990's the PA recommendations also included health-related aspects emphasizing also moderate intensity physical activity with the mention of accumulating physical activity from shorter bouts (≥10 min). These recommendations concluded that adults should accumulate 30 min or more on most days of the week (3). In addition, muscular strength was mentioned for the first time in the recommendations although no specific recommendations were then stated.

Since the early years of the first recommendations, accumulating study results had modified the content and emphasis of the recommendations. The recommendations: “Physical Activity Guidelines for Americans” introduced in 2008 (4) and “Global recommendations on Physical Activity and Health” from WHO introduced in 2010 (5) stated that the minimum recommended amount of PA for adults is aerobic activity either 2 h 30 min per week at moderate-intensity or 1 h and 15 min at vigorous-intensity, which could be accumulated from 10 min activity bouts. In addition, muscular strength and muscular endurance should be performed twice a week. The Finnish physical activity recommendation is based on the “Physical Activity Guidelines for Americans” and was introduced in 2009. In 2018, the “Physical Activity Guidelines for Americans” were revisited. The basic recommendations, mentioned above, stayed the same, however, the borderline of accumulating 10 min activity was excluded and the recommendations states that any activity counts (6).

Considering the accumulating research evidence of beneficial effects of PA from numerous studies during the recent decades, it is surprising how few studies have assessed awareness and knowledge of the recommendations. One of the first studies to assess knowledge of the recommendations was reported by Bennett et al. (7). They reported that 33% of the US adult study sample correctly identified the recommendations for moderate-intensity PA based on recommendations from 1995. Moreover, Moore et al. (8) observed that among 10,000 US adults 26% correctly identified the recommendations from 1995 considering moderate-intensity PA. Moreover, further studies have observed that the knowledge of the recommendations can vary from as less as 1% in a US study population (9) to 8–18% in UK study samples (10–12). In addition to knowledge of PA recommendations, previous studies have found that the proportions of those being aware of the recommendations, varies between 4 and 43% (9, 13–17).

The earlier studies have solely concentrated on studying the knowledge and awareness of the aerobic PA recommendations, whereas the knowledge of muscular type of activity, which is recommended by the present recommendations, have not yet been studied. Therefore, the aim of the present study was to assess awareness and knowledge of the PA recommendations both for aerobic and muscular type of activities. Moreover, the premise for the awareness and knowledge of the physical activity recommendations is that they would optimally be reflected in physical activity behavior. As physical activity is related to improved physical fitness and body composition it is of interest whether these variables are also associated with the awareness and knowledge of the recommendations. The secondary purpose was therefore to study the associations of demographic variables, body mass index (BMI), PA, and physical fitness with awareness, and knowledge of the recommendations. From a national perspective, this is a novel study assessing awareness and knowledge of PA recommendations in a Finnish study sample consisting of young adult men.

## Methods

### Participants

Participants were 776 young (age 26 ± 7 years) adult Finnish men, who were invited in the military refresher training organized by the Finnish Defense Forces. The call up to military refresher training and information about the study plan for participants were sent to participants 5 months before the study conduction, which were carried out in 7 different sessions during 2015. The study protocol was explained in detail to the participants before they gave their written consent. The study was approved by the ethical committees of the University of Jyväskylä and the Central Finland Health Care District, as well as the Headquarters of the Finnish Defense Forces (AM5527). Altogether, 1,106 men were called up and 823 could participate in the military refresher training (response rate 74%). The most typical reasons for non-participation to the military refresher training were related to personal reasons in life, such as work-, study-, or health-related issues. Among those men who participated in the military refresher training 32 refused to take part in the study (response rate 96%). The study sample was compared with corresponding cohorts of 20–30 years old Finnish men in the national register data (Statistics Finland) from the year 2014 for education and place of residence. Based on these analyses, the current study sample can be considered to represent a young adult Finnish man with the following limitations: Northern and Southern Finland were slightly over-represented and the proportion of those participants who had studied 13 years or more was slightly over-represented.

### Assessment of Knowledge and Awareness of Physical Activity Recommendations

Knowledge and awareness of physical activity recommendations were assessed by a questionnaire similar to previous studies (7, 10, 11, 14). To assess awareness of the recommendations participants were asked whether *they have seen, heard or read about physical activity recommendations*? The answer options were: (1) yes, (2) no, and (3) I don't know. For knowledge of the recommendations participants were asked: “*What is the recommended minimum amount of moderate-intensity aerobic physical activity per week based on the present physical activity recommendations.”* The answer options were as follows: (1) 1 h 30 min, (2) 2 h 30 min, (3) 3 h, (4) 20 min per day 3 days per week, (5) 30 min per day 5 days per week, (6) 30 min per day 7 days per week, (7) 60 min per day 7 days per week, (8) none of the previous options, (9) I don't know. Furthermore, *Participants were asked: “What is the recommended minimum amount of muscular strength and endurance type of activity per week based on the present physical activity recommendations.”* The answer options were: (1) once per week, (2) 2 times per week, (3) 3 times per week, (4) 4 times per week, (5) 5 times per week, (6) none of the previous options, (7) I don't know. Moreover, based on the answers given, further classifications were modulated as correct knowledge (answer 2, 5), under estimation (answers 1, 4), overestimation (answers 3, 6, 7) for moderate-intensity aerobic activity knowledge and for muscular type of activity knowledge: correct knowledge (answer 2), under estimation (answers 1), over estimation (answers 3–5). Regarding moderate-intensity aerobic PA, answer option 5 (30 min per day 5 days per week) was classified as correct answer, although it is correct only based on the amount and not by the frequency of the present physical activity recommendations.

### Demographic and Background Variables

Demographic variables were assessed by a questionnaire: age, marital status (married/partnered, divorced/widowed, never married), education (≤9, 10–12, 13–15, ≥16 years), employment status (employed, student, unemployed, or other), and smoking (smoker, quit smoking, non-smoker).

### Assessment of Self-Reported Physical Activity

Leisure-time physical activity (LTPA) was assessed with the following question: “Which of the following definitions best describe your leisure time physical activity habits?”—(“Think of the last 3 months and consider all LTPA that lasted at least 20 min per session”). Response categories were (1) less than once a week; (2) no vigorous activities, but light or moderate physical activity at least once a week (if more often than once please define the numbers per week in an open space); (3), vigorous activity once a week; (4) vigorous activity twice a week; (5) vigorous activity 3 times a week; (6) vigorous activity at least 4 times a week. LTPA was classified as low (responses 1 or 2), moderate (responses 3 or 4), and high (responses 5 or 6) activity (18). The LTPA question used in the present study has been validated against fitness, observing that vigorous physical activity showed a consistent dose-response relationship with cardiorespiratory and muscular fitness (19). In general, acceptable to good reliability but poor to moderate validity has been reported for physical activity questionnaires (20).

### Physical Fitness

The participants performed the physical fitness tests in the following order: standing long jump, isometric maximal force, cardiorespiratory fitness, and muscular endurance tests. All of the participants were familiar with the standing long jump test and muscular endurance tests because those tests have been conducted during their military service.

#### Standing Long Jump Test

*Standing long jump test* was used to test explosive force production of lower extremities (21). The participants were instructed of the correct technique prior the testing, and each of them performed several jumps after a warm-up and prior the testing, which was performed in the specifically designed gym mat. The warm-up lasted 10 min and consisted of calisthenics exercises, such as x-jumps, push-ups, sit-ups, squats, planks, and countermovement jumps. The participants were instructed to jump (horizontally) forward as far as possible from a standing position, using a prior countermovement and hands freely swinging by their sides without falling backward upon bilateral landing. The participants completed 3 trials each interspersed by a 1-min rest period. The performance was measured with 1 cm precision.

#### Maximal Isometric Force

*Maximal isometric force* was measured with horizontal bench press (both regarded as tests for maximal strength) using a dynamometer. Knee angle was set to 107° with a goniometer, and hands were placed on a handle grip in a leg extension test (22). During the maximal bench press test, the participants were in supine position with their backs flat on a bench and feet flat on the floor with elbow and shoulder positioned at 90°. A warm-up series of at least 2 submaximal sets were done prior to maximal sets. Three trials were performed using a 30-s recovery period. The best performance was included in the analysis. Each participant was advised to produce maximal force as fast as possible and to maintain it for 3 s. The participants were verbally encouraged during the maximal efforts by the test personnel. The repeatability has been reported to be high in maximal isometric strength tests (*r* = 0.98, C.V = 4.1%) (23).

#### Cardiorespiratory Fitness

*Cardiorespiratory fitness* (VO_2_ max) was determined using an indirect graded cycle ergometer test (Ergoline 800S, Ergoselect 100K, Ergoselect 200K, Bitz, Germany) until exhaustion. A progressive protocol was used, which initially started at a power output of 50 W and was increased 25 W every 2 min until exhaustion. Heart rate (HR) was continuously recorded during the test (Polar Vantage NV or S610, S710, or S810, Kempele, Finland). Predicted VO_2_ max was estimated from HR and maximal power (W) (Fitware, Mikkeli, Finland) with the following equation: VO_2_ max (ml·kg^−1^·min^−1^) = 12.35 × Pmax/kg + 3.5, where Pmax is maximal power and kg is body mass in kg. The intra class correlation has been reported to be high with this method (24).

#### Muscular Endurance Tests

*Muscular endurance tests* consisted of push-ups and sit-ups (repetitions/minute). The push-up test measured the performance of arm and shoulder extensor muscles. Before taking a starting position, the participants laid face down on the floor, feet parallel at pelvis to shoulder width and hands positioned so that thumbs could reach the shoulders while other fingers were pointing forward. Before the beginning of the test, the participants were instructed to extend their arms to the starting position and keep the feet, trunk, and the shoulders in the same line throughout the test. One successful repetition was counted when the participant lowered his torso by flexing arms to an elbow angle of 90° and returned to the starting position by extending his arms. Sit-up test was used to measure performance of abdominal and hip flexor muscles. In the starting position, the participants laid on his back while legs were supported from the ankles by an assistant. The knees were flexed at the angle of 90°, elbows pointing upward and fingers crossed behind the back of the head. One successful repetition was determined when the participant lifted his upper body from the starting position and brought elbows to the knee-level. The result of the test was expressed as a number of consecutive successful repetitions during 60 s. There was a recovery period of 5-min between the tests. Correct technique was demonstrated to participants before each test and only the trials with adequate technique were accepted. The test-retest reliability of push-up, sit-up, and repeated squat tests has been reported to be high among young adults and middle-aged adults (ICC = 0.93–0.95, ICC = 0.83–0.93, *r* = 0.95, respectively) (25, 26).

### Body Composition

Body weight was measured to the nearest 0.1 kg and body height by a commercial scale to the nearest 0.1 cm, and further BMI was calculated.

### Statistical Analysis

Data was analyzed with IBM SPSS Statistics 22.0.0.0. Descriptive statistics as frequencies, means, standard deviations and 95% confidence intervals were calculated and are presented in the tables. χ^2^ -tests were used to explore the associations of marital status, employment status, smoking, and PA with the awareness and knowledge of the recommendations. For continuous variables age, BMI, and physical fitness tests, the difference of means in awareness and knowledge of recommendations were assessed with analysis of variance using Bonferroni *post hoc* tests. Moreover, those parameters that were not normally distributed were log-transformed (maximal isometric force of upper and lower extremities).

## Results

The background characteristics of the study participants is presented in the Table 1. Forty percent of the participants reported being aware and 27% not being aware of the PA recommendations. Table 2 reveals that 7% correctly identified the recommendation for moderate aerobic PA and 25% for muscular type of activity. The proportion of those who correctly identified both aerobic and muscular activity recommendations was 4% (*n* = 31). In addition, 16% overestimated and 25% underestimated the recommended aerobic PA and 3% underestimated and 37% overestimated the recommended muscular type of activity.

**Table 1:** Demographic characteristics (%), physical activity (%), and fitness (mean ± SD) of the study participants.

**Characteristics**			***n***
Age (years)		26 ± 7	767
Marital status (%)	Married/partnered	44.5	339
	Divorced/widowed	3.3	25
	Never married	52.2	398
Education (%)	≤9 years	3.3	25
	10–12 years	34.0	259
	13–15 years	41.9	319
	≥16 years	20.9	159
Employment status (%)	Employed	69.2	527
	Student	24.7	188
	Unemployed or other	6.2	47
Smoking (%)	Smokers	26.1	199
	Quit smoking	24.4	186
	Non-smokers	49.0	377
Body mass index (%)	Underweight (<18.5)	2.1	16
	Normal weight (18.5–24.99)	51.5	388
	Overweight (25–29.99)	34.1	257
	Obese (≥30)	12.3	93
Physical activity (%)	Low	29.9	228
	Moderate	29.8	227
	High	40.3	307
Physical fitness	VO_2_ max (ml/kg/min)	41.1 ± 7.8	740
	Standing long jump (cm)	226.6 ± 26.2	742
	Sit-ups (reps/min)	34.9 ± 11.9	741
	Push-ups (reps/min)	28.4 ± 13.9	739
	Maximal strength in bench press (*N*)	871 ± 216	745
	Maximal strength in leg extension (*N*)	3394 ± 933	744

**Table 2:** Distributions of answers regarding awareness and knowledge of the physical activity recommendations.

	**%**	***n***
**I have seen, heard or read about physical activity recommendations for adults**		
Yes	39.5	300
No	26.6	202
I don't know	33.9	257
**Moderate aerobic physical activity recommendation per week is:**		
1 h 30 min	3.6	28
2 h 30 min	6.9	54
3 h	12.0	94
20 min per day 3 days per week	21.8	170
30 min per day 5 days per week	15.0	117
30 min per day 7 days per week	3.6	28
60 min per day 7 days per week	0.6	5
None of the previous options	1.3	10
I don't know	35.2	275
**Muscular fitness recommendation per week is:**		
Once per week	2.9	22
2 times per week	25.1	189
3 times per week	29.1	219
4 times per week	6.6	50
5 times per week	2.0	15
None of the previous options	0.9	7
I don't know	33.3	251

Marital status (married/partnered), education level and leisure-time PA were positively associated with awareness of the recommendations (Table 3). Table 3 further demonstrates that those individuals with no awareness of the PA recommendations had lower results in cardiorespiratory fitness and sit-ups compared to those individuals being aware (*p* < 0.05) or those who answered don't know (*p* < 0.05). Furthermore, individuals with no awareness of the PA recommendations had lower results in push-ups and maximal strength of upper extremities compared to those individuals being aware (*p* < 0.05).

**Table 3:** The distributions and mean ± sd of those of being aware, not aware and those who don't know of the physical activity recommendations according to demographic variables, body composition, and physical activity and fitness.

**Variable** ** (main effect)**		**Aware (%)** ** (39.5%)**	**Not aware (%)** ** (26.6%)**	**Don't know (%)** ** (33.9%)**
**Age (years)** (*p* = 0.223)		27.4 ± 7.5	26.3 ± 6.5	25.2 ± 5.7
**Marital status**	Married/partnered	52.0 (*n* = 156)	39.6 (*n* = 80)	40.1 (*n* = 103)
(*p* = 0.012)^*^	Divorced/widowed	3.7 (*n* = 11)	4.0 (*n* = 8)	2.3 (*n* = 6)
	Never married	44.3 (*n* = 133)	56.4 (*n* = 114)	57.6 (*n* = 148)
**Education**	≤9 years	3.7 (*n* = 11)	1.0 (*n* = 2)	4.7 (*n* = 12)
(*p* = 0.009)^*^	10–12 years	27.3 (*n* = 82)	36.6 (*n* = 74)	39.3 (*n* = 101)
	13–15 years	43.7 (*n* = 131)	43.1 (*n* = 87)	38.9 (*n* = 100)
	≥16 years	25.3 (76)	19.3 (*n* = 39)	17.1 (*n* = 44)
**Employment status**				
(*p* = 0.811)	Employed	70.0 (*n* = 210)	71.8 (*n* = 145)	66.5 (*n* = 171)
	Student	24.0 (*n* = 72)	22.8 (*n* = 46)	26.8 (*n* = 69)
	Unemployed	6.0 (*n* = 18)	5.4 (*n* = 11)	6.6 (*n* = 17)
**Smoking**	Smokers	27.3 (*n* = 82)	25.7 (*n* = 52)	24.5 (*n* = 63)
(*p* = 0.564)	Quit smoking	22.3 (*n* = 67)	22.8 (*n* = 46)	28.0 (*n* = 72)
	Non-smokers	50.3 (*n* = 151)	51.5 (*n* = 104)	47.5 (*n* = 122)
**Body mass index**	Underweight (<18.5)	2.7 (*n* = 8)	2.0 (*n* = 4)	1.6 (*n* = 4)
(*p* = 0.491)	Normal weight (18.5–24.99)	48.5 (*n* = 142)	54.5 (*n* = 109)	52.8 (*n* = 133)
	Overweight (25–29.99)	36.2 (*n* = 106)	29.0 (*n* = 58)	35.5 (*n* = 89)
	Obese (≥30)	12.6 (*n* = 37)	14.5 (*n* = 29)	10.3 (*n* = 26)
**Leisure-time physical activity**				
(*p* = 0.001)^***^	Low	26.0 (*n* = 78)	36.6 (*n* = 74)	29.2 (*n* = 75)
	Moderate	24.7 (*n* = 74)	32.2 (*n* = 65)	33.9 (*n* = 87)
	High	49.3 (*n* = 148)	31.2 (*n* = 63)	37.0 (*n* = 95)
**Physical fitness**				
(*p* = 0.017)^*^	VO_2_ max (ml/kg/min)	41.5 ± 8.1	39.7 ± 7.9^*^	41.7 ± 7.4 ≠
(*p* = 0.247)	Standing long jump (cm)	228.4 ± 26.5	224.3 ± 27.1	226.6 ± 25.3
(*p* ≤0.001)^***^	Sit-ups (reps/min)	36.9 ± 11.5	32.4 ± 12.1^***^	34.5 ± 11.8 ≠
(*p* = 0.027)^*^	Push-ups (reps/min)	30.0 ± 14.2	26.6 ± 12.9^*^	28.0 ± 14.1
(*p* = 0.009)^*^	Maximal strength in bench press (N)	903 ± 237	841 ± 210^*^	858 ± 190
(*p* = 0.067)	Maximal strength in leg extension (N)	3503 ± 988	3312 ± 868	3333 ± 896

* p < 0.05 statistically significant trend between the subgroups ^***^p < 0.001 statistically significant trend between the subgroups.

For physical fitness variables:

*** p < 0.001 compared to “aware subgroup,” ^*^p < 0.05 compared to “aware subgroup,” ≠ p < 0.05 compared to “not aware subgroup”.

Marital status (married/partnered) was positively associated with the knowledge of muscular type of activity recommendations (**Table 5**) but not with moderate aerobic PA recommendations (Table 4). Regarding knowledge of moderate aerobic PA recommendations, those who responded “don‘t know” had lower results in standing long jump compared to those individuals with correct knowledge (*p* < 0.05) and lower results in sit-ups compared to those individuals with correct knowledge (*p* < 0.05) and those who overestimated the PA recommendations (*p* < 0.05) (Table 4).

**Table 4:** The distributions and mean ± sd of correct, unsure, and over- and under-estimation of the moderate aerobic physical activity recommendations according to demographic variables, body composition, and physical activity and fitness.

**Variable** ** (main effect)**		**Correct** ** knowledge** ** (*n* = 22.2%)**	**Under-estimation** ** (25.3%)**	**Over-** **estimation** ** (15.6%)**	**Don't** ** know** ** (*n* = 36.9%)**
**Age (years)** (*p* = 0.078)		26.4 ± 6.8	27.8 ± 8.5	26.8 ± 7.6	25.9 ± 5.7
**Marital status**	Married/partnered	45.0 (*n* = 76)	44.6 (*n* = 86)	47.1 (*n* = 56)	43.1 (*n* = 121)
(*p* = 0.822)	Divorced/widowed	4.1 (*n* = 7)	3.6 (*n* = 7)	4.2 (*n* = 5)	2.1 (*n* = 6)
	Never married	50.9 (*n* = 86)	51.8 (*n* = 100)	48.7 (*n* = 58)	54.8 (*n* = 154)
**Education**	≤9 years	4.1 (*n* = 7)	4.1 (*n* = 8)	2.5 (*n* = 3)	2.5 (*n* = 7)
(*p* = 0.854)	10–12 years	29.6 (*n* = 50)	34.2 (*n* = 66)	37.0 (*n* = 44)	35.2 (*n* = 99)
	13–15 years	46.2 (*n* = 78)	42.5(*n* = 82)	38.7 (*n* = 46)	40.2 (*n* = 113)
	≥16 years	20.1 (*n* = 34)	19.2 (*n* = 37)	21.8 (*n* = 26)	22.1 (*n* = 62)
**Employment status**					
(*p* = 0.451)	Employed	65.7 (*n* = 111)	69.9 (*n* = 135)	76.5 (*n* = 91)	67.6 (*n* = 190)
	Student	29.0 (*n* = 49)	22.8 (*n* = 44)	18.5 (*n* = 22)	26.0 (*n* = 73)
	Unemployed	5.3 (*n* = 9)	7.3 (*n* = 14)	5.0 (*n* = 6)	6.4 (*n* = 18)
	Smokers	50.3 (*n* = 85)	47.2 (*n* = 91)	52.1 (*n* = 62)	49.5 (*n* = 139)
**Smoking**	Quit smoking	21.3 (*n* = 36)	23.3 (*n* = 45)	24.4 (*n* = 29)	27.0 (*n* = 76)
(*p* = 0.648)	Non-smokers	28.4 (*n* = 48)	29.5 (*n* = 57)	23.5 (*n* = 28)	23.5 (*n* = 66)
**Body mass index**	Underweight (<18.5)	2.4 (*n* = 4)	1.6 (*n* = 3)	2.6 (*n* = 3)	2.2 (*n* = 6)
(*p* = 0.298)	Normal weight (18.5–24.99)	57.9 (*n* = 95)	48.7 (*n* = 93)	41.4 (*n* = 48)	53.6 (*n* = 148)
	Overweight (25–29.99)	27.4 (*n* = 45)	37.7 (*n* = 72)	43.1 (*n* = 50)	31.9 (*n* = 88)
	Obese (≥30)	12.2 (*n* = 20)	12.0 (*n* = 23)	12.9 (*n* = 15)	12.3 (*n* = 34)
**Leisure-time physical activity**					
(*p* = 0.076)	Low	18.9 (*n* = 43)	27.2 (*n* = 62)	11.4 (*n* = 26)	42.5 (*n* = 97)
	Moderate	22.5 (*n* = 51)	22.0 (*n* = 50)	19.8 (*n* = 45)	35.7 (*n* = 81)
	High	24.4 (*n* = 75)	26.4 (*n* = 81)	15.6 (*n* = 48)	33.6 (*n* = 103)
**Physical fitness**					
(*p* = 0.828)	VO_2_ max (ml/kg/min)	42.3 ± 7.9	42.4 ± 7.9	40.7 ± 7.4	40.3 ± 7.8
(*p* = 0.018)^*^	Standing long jump (cm)	229.1 ± 25.5	228.5 ± 26.7	227.4 ± 25.1	223.4 ± 28.0^*^
(*p* = 0.002)^*^	Sit-ups (reps/min)	38.1 ± 11.8	35.2 ± 13.1	34.5 ± 11.4	33.0 ± 11.9^*^^†^
(*p* = 0.061)	Push-ups (reps/min)	30.8 ± 13.6	27.4 ± 12.4	29.3 ± 14.3	25.8 ± 13.5
(*p* = 0.070)	Maximal strength in bench press (N)	913 ± 22.2	814 ± 13.3	891 ± 227	830 ± 20
(*p* = 0.172)	Maximal strength in leg extension (N)	3495 ± 1003	3118 ± 878	3434 ± 968	3297 ± 805

* p < 0.05 statistically significant trend between the subgroups. For physical fitness variables: ^*^p < 0.05 compared to “correct knowledge” subgroup,

† *p < 0.05 compared to “over-estimation” subgroup*.

Regarding knowledge of muscular type of PA recommendations, those who responded “don't know” had lower results in push-ups and maximal strength of upper extremities compared to those individuals with correct knowledge (*p* < 0.05) and those who overestimated the PA recommendations (*p* < 0.05) (Table 5). Moreover, those individuals who responded “don't know” and those who overestimated the muscular type of PA recommendations had lower results in sit-ups compared to individuals with correct knowledge (*p* < 0.05) (Table 5).

**Table 5:** The distributions and mean ± sd of correct, unsure, and over- and under-estimation of the muscular strength and muscular endurance recommendations according to demographic variables, body composition, and physical activity and fitness.

**Variable** ** (main effect)**		**Correct** ** knowledge** ** (25.2%)**	**Under-** **estimation** ** (2.8%)**	**Over-** **Estimation** ** (37.4%)**	**Don't** ** know/none of these** ** (34.6%)**
**Age (years)** (*p* = 0.830)		26.4 ± 6.8	27.8 ± 8.5	26.8 ± 7.6	25.9 ± 5.7
**Marital status**	Married/partnered	50.0 (*n* = 93)	57.1 (*n* = 12)	42.8 (*n* = 118)	42.4 (*n* = 108)
(*p* = 0.016)^*^	Divorced/widowed	3.8 (*n* = 7)	14.3 (*n* = 3)	3.6 (*n* = 10)	2.0 (*n* = 5)
	Never married	46.2 (*n* = 86)	28.6 (*n* = 6)	53.6 (*n* = 148)	55.7 (*n* = 142)
**Education**	≤9 years	3.2 (*n* = 6)	9.5 (*n* = 2)	4.3 (*n* = 12)	2.0 (*n* = 5)
(*p* = 0.314)	10–12 years	32.3 (*n* = 60)	42.9 (*n* = 9)	34.1 (*n* = 94)	33.7 (*n* = 86)
	13–15 years	39.8 (*n* = 74)	33.3 (*n* = 7)	44.6 (*n* = 123)	41.6 (*n* = 106)
	≥16 years	24.7 (*n* = 46)	14.3 (*n* = 3)	17.0 (*n* = 47)	22.7 (*n* = 58)
**Employment status**					
(*p* = 0.151)	Employed	66.7 (*n* = 124)	81.0 (*n* = 17)	73.9 (*n* = 204)	64.7 (*n* = 165)
	Student	28.0 (*n* = 52)	9.5 (*n* = 2)	20.3 (*n* = 56)	27.8 (*n* = 71)
	Unemployed	5.4 (*n* = 10)	9.5 (*n* = 2)	5.8 (*n* = 16)	7.5 (*n* = 19)
**Smoking**	Smokers	23.7 (*n* = 44)	28.6 (*n* = 6)	31.2 (*n* = 86)	22.7 (*n* = 58)
(*p* = 0.141)	Quit smoking	21.5 (*n* = 40)	14.3 (*n* = 3)	25.4 (*n* = 70)	25.9 (*n* = 66)
	Non-smokers	54.8 (*n* = 102)	57.1 (*n* = 12)	43.5 (*n* = 120)	51.4 (*n* = 131)
**Body mass index**	Underweight (<18.5)	0.6 (*n* = 1)	9.5 (*n* = 2)	3.3 (*n* = 9)	1.2 (*n* = 3)
(*p* = 0.079)	Normal weight (18.5–24.99)	54.1 (*n* = 98)	52.4 (*n* = 11)	45.6 (*n* = 12.4)	53.8 (*n* = 134)
	Overweight (25–29.99)	34.3 (*n* = 62)	23.8 (*n* = 5)	38.2 (*n* = 104)	32.1 (*n* = 80)
	Obese (≥30)	11.0 (*n* = 20)	14.3 (*n* = 3)	12.9 (*n* = 35)	12.9 (*n* = 32)
**Leisure-time physical activity**					
	Low	25.8 (*n* = 48)	23.8 (*n* = 5)	28.6 (*n* = 79)	35.3 (*n* = 90)
(*p* = 0.356)	Moderate	32.3 (*n* = 60)	28.6 (*n* = 6)	28.6 (*n* = 79)	29.0 (*n* = 74)
	High	41.9 (*n* = 78)	47.6 (*n* = 10)	42.8 (*n* = 118)	35.7 (*n* = 91)
**Physical fitness**					
(*p* = 0.971)	VO_2_ max (ml/kg/min)	42.3 ± 7.9	42.4 ± 7.9	40.7 ± 7.4	40.2 ± 7.8
(*p* = 0.138)	Standing long jump (cm)	229.1 ± 25.5	228.5 ± 26.7	227.4 ± 25.1	223.4 ± 28.0
(*p* <0.001)^***^	Sit-ups (reps/min)	38.1 ± 11.8	35.2 ± 13.1	34.5 ± 11.4^*^	33.0 ± 11.9^***^
(*p* = 0.002)^*^	Push-ups (reps/min)	30.8 ± 13.6	27.4 ± 12.4	29.3 ± 14.3	25.8 ± 13.5^*^^†^
(*p* <0.001)^***^	Maximal strength in bench press (N)	913 ± 222	813 ± 133	891 ± 227	830 ± 200^***^^†^
(*p* = 0.087)	Maximal strength in leg extension (N)	3495 ± 1003	3118 ± 878	3434 ± 968	3297 ± 805

* p < 0.05 statistically significant trend between the subgroups

*** p < 0.001 statistically significant trend between the subgroups. For physical fitness variables: ^*^p < 0.001 compared to “correct knowledge” subgroup, ^*^p < 0.05 compared to “correct knowledge” subgroup,

† *p < 0.05 compared to “over-estimation” subgroup*.

## Discussion

Although 40% of the young adult Finnish men reported being aware of the present PA recommendations only 7% correctly identified the recommendation for moderate aerobic PA and 25% for muscular type of activity recommendation. Moreover, 4% correctly identified both moderate intensity aerobic and muscular type of activity recommendations. Marital status, education, leisure-time PA, and selected fitness variables were positively associated with awareness, whereas selected fitness variables were positively associated with correct knowledge of both aerobic and muscular PA recommendations.

The present study results showed higher awareness of the recommendations compared to most of the previous studies, where the prevalence rates have ranged from 4 to 37% (9, 13, 15). In addition, the previous studies have observed associations of age (9, 13, 16), education (9, 13, 15), gender (9, 13, 15), PA level (13), and ethnicity (9) with awareness. These previous studies do, therefore, suggest that when planning to develop awareness of PA recommendations a specific effort may be needed to target individuals in different subgroups. The present study suggests that inactive or unfit individuals, those with low education level and those not being married or partnered could be specific target groups for PA promotion compared to their counterparts among young adult men. However, it should be noted that on average, considering the present and earlier studies, there are far more individuals that are not aware of the recommendations compared to those who are aware and therefore, promotion of awareness should be vigorously attempted to whole population within its subgroups. Although professionals working with PA promotion generally have higher awareness of the recommendations than rest of the population one previous study (17) has revealed rather disappointing awareness among professionals (34%). Therefore, one of the priorities in raising awareness should also be to educate professionals as well.

Interestingly, despite the awareness of 40% of the present study sample, only 7% correctly identified moderate aerobic recommendations and 25% muscular type of activity recommendations. Nevertheless, if previous activity recommendations from 1995 (3) regarding aerobic PA was to be approved as a correct identification (30 min, 5 times per week) this proportion would have increased to 22%. Previous studies have shown highly varied prevalence of correct knowledge of the moderate aerobic PA recommendations ranging from <1 to 36% (7–12). In the present study, significant associations were observed between selected fitness variables and knowledge for both aerobic and muscular type of physical activity recommendations. In addition, marital status was related to knowledge of muscular type of activity recommendations. These results suggest that married individuals and individuals with higher fitness tend to know recommendations better than non-married individuals and individuals with lower fitness. Nevertheless, no causal relationships can be interpreted because of the cross-sectional study design. Therefore, it remains unknown whether the aware individuals have higher fitness because of the awareness or are they aware because of their higher fitness (a proxy for possibly higher interest in physical activity and fitness). Earlier studies have shown that age (11, 12, 14), education (9, 11, 12, 14), gender (7, 8, 13, 14), physical activity level (7, 8, 12, 13), ethnicity (8), income (8, 9, 12, 14), employment status (7, 11), and self-rated health (12) are associated with knowledge of the recommendations.

A higher number of individuals correctly identified muscular type of activity (25%) compared to moderate intensity aerobic activity (7%). This may be due to different answer options given or a speculatively a tendency of young adult men to be more interested in resistance training and its recommendations than aerobic exercise and recommendations. Nevertheless, if previous moderate aerobic PA recommendations would have been accepted as a correct alternative, based on its equal amount of activity (30 min, 5 times per week) altogether 22% of the participants would have been reported correct amount of moderate aerobic PA. Using this compound as a correct knowledge of aerobic activity recommendations we found that 22% of the study sample had correct knowledge, 25% underestimated, and 27% overestimated the moderate aerobic activity recommendations. The respective proportions for muscular type of activity recommendations were 22, 25, and 27%. Again, differences in given answer options in aerobic and muscular fitness questions may have affected the results because in the muscular type of activity question only exercise frequency was assessed, whereas aerobic activity question included different frequencies and volumes. The different assessment methods previous studies makes it hard to draw definite collective conclusions about the knowledge of the recommendations. However, of importance, some of the previous studies (8, 9, 12, 14) including the present study, have used prompted questionnaire, which may overestimate the prevalence of correct knowledge, whereas some other studies have used unprompted questionnaires (7, 10, 11, 14). The overestimation has been observed in a study by Cameron et al. (14), where they reported a significant difference between prompted and unprompted questions showing that the correct knowledge decreased from 37 to 4% when prompted and unprompted question format was compared. Therefore, the prevalence of knowledge of the earlier and the present study may actually be even less than reported given that prompted format was used.

A few practical conclusions regarding awareness and knowledge of the PA recommendations can be made based on the present and previous study findings. Firstly, based on low awareness and, especially, poor knowledge of the recommendations all attempts to raise awareness and knowledge should be made. Previous studies show that different methods to deliver health messages and health behavior change may work (27). The fact that the effect size of e.g., mass media campaigns on health behavior has turn out to be rather low emphasizes the need for multiple methods to raise awareness and knowledge. Besides community-wide campaigns, tools for dissemination could include e.g., social marketing through social media, online advertising and delivery of print products (28–30).

The strengths of the present study are a nationally representative study population targeted to young adult men. In addition, to the best of our knowledge, this is the first study that has investigated the knowledge of muscular type of activity recommendations. There are, however, some limitations in the current study. As noted earlier, prompted questionnaires were used in the present study, which may overestimate the proportions of knowledge and awareness individuals. Secondly, the present study did only assess knowledge of moderate-intensity aerobic physical activity recommendations and not vigorous aerobic physical activity. Therefore, future studies are warranted to assess all components of the present recommendations.

## Conclusions

In conclusion, the present study revealed that 40% of the young adult Finnish men reported being aware of the present PA recommendations, however, only 7% correctly identified the recommendation for moderate aerobic activity and 25% for muscular fitness type of activity. Altogether, 4% correctly identified both moderate intensity aerobic and muscular type of activity recommendations. Marital status, education, LTPA, and selected fitness variables were positively associated with awareness, while selected fitness variables were positively associated with correct knowledge of both aerobic and muscular strength recommendations. These results underline a need to use multiple methods to raise awareness and knowledge of the physical activity recommendations among young adults.

## Data Availability Statement

All datasets generated for this study are included in the article/supplementary material.

## Ethics Statement

The studies involving human participants were reviewed and approved by the study was approved by the ethical committees of the University of Jyväskylä and the Central Finland Health Care District, as well as the Headquarters of the Finnish Defense Forces. The patients/participants provided their written informed consent to participate in this study.

## Author Contributions

JV performed experiments and analyzed data. JV, TV, HKo, and HKy conceived and designed the experiments, interpreted results of research, drafted, edited, critically revised paper, and approved final version of manuscript.

### Conflict of Interest

The authors declare that the research was conducted in the absence of any commercial or financial relationships that could be construed as a potential conflict of interest.
